# Swine Workers and Swine Influenza Virus Infections

**DOI:** 10.3201/eid1312.061323

**Published:** 2007-12

**Authors:** Gregory C. Gray, Troy McCarthy, Ana W. Capuano, Sharon F. Setterquist, Christopher W. Olsen, Michael C. Alavanja, Charles F. Lynch

**Affiliations:** * University of Iowa College of Public Health, Iowa City, Iowa, USA; † University of Wisconsin-Madison School of Veterinary Medicine, Madison, Wisconsin, USA; ‡ National Cancer Institute, Bethesda, Maryland, USA

**Keywords:** Influenza, influenza A virus, porcine, zoonoses, occupational exposure, communicable diseases, emerging, agriculture, seroepidemiologic studies, disease outbreaks, epidemiology, research

## Abstract

In 2004, 803 rural Iowans from the Agricultural Health Study were enrolled in a 2-year prospective study of zoonotic influenza transmission. Demographic and occupational exposure data from enrollment, 12-month, and 24-month follow-up encounters were examined for association with evidence of previous and incident influenza virus infections. When proportional odds modeling with multivariable adjustment was used, upon enrollment, swine-exposed participants (odds ratio [OR] 54.9, 95% confidence interval [CI] 13.0–232.6) and their nonswine-exposed spouses (OR 28.2, 95% CI 6.1–130.1) were found to have an increased odds of elevated antibody level to swine influenza (H1N1) virus compared with 79 nonexposed University of Iowa personnel. Further evidence of occupational swine influenza virus infections was observed through self-reported influenza-like illness data, comparisons of enrollment and follow-up serum samples, and the isolation of a reassortant swine influenza (H1N1) virus from an ill swine farmer. Study data suggest that swine workers and their nonswine-exposed spouses are at increased risk of zoonotic influenza virus infections.

## Abstract 2

Swine workers and their spouses are at markedly increased risk of acquiring swine influenza virus infections.

Since 1997, numerous instances of avian influenza virus infection have been documented in humans (*1*). The latest of such viruses, strains of subtype H5N1, have rapidly spread among domestic bird species across several continents and caused disease in >330 humans since 2003 (*2*). Like the influenza (H5N1) viruses that are circulating today, a highly virulent avian virus subtype, H1N1, was responsible for the 1918–1919 pandemic. Coincident with the human pandemic, this virus also infected swine, caused large-scale epizootics of swine respiratory disease in the midwestern United States, and established itself among pigs as the “classical” swine influenza virus lineage of influenza (H1N1) viruses (*3*,*4*). It also apparently moved from swine to humans, causing illness among farmers (*3*). Anticipating that the next pandemic virus may similarly be readily transmitted among and between pigs and humans, we sought to prospectively study swine workers for risk factors for swine influenza virus infection.

## Methods

### Study Population

After institutional review board approval, participants were recruited from the 89,658-person Agricultural Health Study (AHS) cohort (*5*) by using an informed consent process. The cohort, first assembled from 1993 through 1997, comprises primarily private pesticide applicators (predominately farmers) and their spouses living in Iowa and North Carolina. Through a stratified sampling scheme, participants living in Iowa were selected by previously reported exposures to swine or poultry, age group, sex, and proximity to the University of Iowa in Iowa City. Nonswine- and nonpoultry-exposed potential participants were similarly selected.

Potential AHS participants and their spouses were screened by telephone interviews and verified to be without immunocompromised conditions and without a history of accidental injection with swine influenza vaccines. They were then invited to participate in a 2-year prospective study of zoonotic influenza transmission. Enrollments were made through personal interviews held in 29 of the 99 counties in Iowa during the fall of 2004. After informed consent was obtained, each participant completed a questionnaire and permitted serum sample collection. Swine exposure was assessed by the participant’s response to the enrollment question: “How many years have you worked in swine production?” Participants who answered “never” were classified as nonexposed. Follow-up visits with similar questionnaires and phlebotomy were scheduled at 12 and 24 months. Upon enrollment and at 12 months, participants were given a first-class US Postal Service–ready kit with detailed instructions to complete another questionnaire and self-collect gargle and nasal swab specimens within 96 h of symptom onset if they met a case definition of influenza-like illness (fever >38°C and a cough or sore throat). The kit contained a freezer block that participants were asked to insert into the preaddressed shipping box before dropping off specimens and questionnaires with the US Postal Service. The US post office near the University of Iowa laboratory kept these boxes refrigerated until the study team picked them up on regular work days.

Data and serum samples from nonagricultural health study controls from a concurrent cross-sectional study (*6*) were included in population comparisons at enrollment. Study controls were generally healthy University of Iowa students, staff, and faculty who denied having swine or poultry exposures. They were not studied at 12 and 24 months after enrollment.

### Laboratory Methods

#### Specimens

Gargle and swab specimens were transported to the University of Iowa by the US Postal Service in Micro Test M4RT Viral Transport Media (Remel, Inc., Lenexa, KS, USA) and preserved at –80°C. These specimens were studied with both culture in MDCK cells and R-Mix FreshCells (Diagnostic Hybrids, Inc., Athens, OH, USA) and with molecular techniques.

#### Hemagglutination-Inhibition (HI) Assay

Per our previous reports (*6*,*7*), serum samples were tested by using Centers for Disease Control and Prevention (CDC) HI assay protocol against 4 isolates of recently circulating swine and human influenza A viruses: A/swine/WI/238/97 (H1N1), A/swine/WI/R33F/2001 (H1N2), A/New Caledonia/20/99 (H1N1), and A/Panama/2007/99 (H3N2). Swine virus isolates were selected and provided by one of the authors (C.O.). A/swine/WI/238/97 (H1N1) is a classic swine (H1N1) virus (*8*). A/swine/WI/R33F/2001 (H1N2) is representative of reassortant (H1N2) viruses with classic swine virus HA, M, NP, and NS genes, human virus NA and PB1 genes, and avian virus PA and PB2 genes that first appeared among US pigs in 1999 (*9*,*10*).

The human viral strains and the A/swine/WI/238/97 swine strain were grown in embryonated chicken eggs; the A/swine/WI/R33F/2001 strain was grown in MDCK cells. Serum samples were pretreated with receptor destroying enzyme per CDC protocol. Prior to serum HI testing for the human strains, samples were hemabsorbed with guinea pig erythrocytes. A second aliquot of receptor-destroying enzyme-treated serum was hemabsorbed with turkey erythrocytes before HI testing of the swine strains. Titer results are reported as the reciprocal of the highest dilution of serum that inhibited virus-induced hemagglutination of a 0.65% (guinea pig) or 0.50% (turkey) solution of erythrocytes.

### Molecular Studies

#### Real-time Reverse Transcription–PCR (RT-PCR)

RNA was extracted from 140 μL of each nasal swab and gargle sample using a QIAamp viral RNA extraction kit (QIAGEN Inc., Valencia, CA, USA) and screened by using a proprietary real-time RT-PCR protocol developed and provided by CDC. CDC’s protocol is designed to first screen for influenza A, and then, through separate reactions, to rapidly determine influenza HA subtype. iScript One-Step RT-PCR Kit for Probes (Bio-Rad, Hercules, CA, USA) and the iQ Real-Time PCR Detection System (Bio-Rad) were used on a Bio-Rad iCycler real-time PCR platform for the real-time RT-PCR. Negative template controls and positive controls were included on each run. The human RNase P gene served as an internal control for human RNA. Clinical samples with negative results for the RNAse P gene were repeated. Samples positive by real-time RT-PCR for influenza A were further studied with RT-PCR and cDNA sequencing for phylogenetic analyses to confirm subtype and, in some cases, for further genotypic analyses, using previously described techniques and primers (*9*–*14*).

#### Cross-reactivity and Reliability

As we had previously identified partial serologic cross-reactivity between swine and human viral strains of the same hemagglutinin types (*6*), we adjusted for this potential confounding in each of the risk factor analyses by including human serologic results in the models. Regarding laboratory assay reliability, our previous study found 80% and 70% agreement (within 1 titer) for repeat swine influenza (H1N2) and (H1N1) virus testing, respectively (*6*).

### Statistical Methods

We examined a number of potential risk factors for association with influenza virus infection outcomes: sex, age, influenza vaccination (human) history, seropositivity for human influenza viruses, years in swine production, days per week working with swine, use of personal protective equipment, recent swine exposure, number of pigs on the farm, and type of swine farm. HI test results from enrollment serum samples were first dichotomized with titers >40 considered as evidence of previous infection (*15*,*16*). The χ^2^ statistic or 2-sided Fisher exact test was used to examine bivariate risk factor associations. Age was examined by using analysis of variance. Geometric mean HI titers were calculated for each virus strain. Titer distribution was compared with potential risk factors by using the Wilcoxon rank-sum test with normal approximation. Afterwards, the distribution of antibody titer levels was examined for associations with multiple risk factors by using both unconditional logistic regression and proportional odds modeling (*17*). The score test was used to evaluate the proportional odds assumption. Final multivariable models were designed by using a saturated model including all potential risk factors and manual backwards elimination. Analyses were performed by using SAS software version 9.1 (SAS Institute, Inc., Cary, NC, USA).

We used bivariate and unconditional logistic regression to examine risk factors for evidence of influenza virus infection in 2 ways. First, using the classical approach, we examined risk factor associations for any 4-fold rise in HI titer (enrollment to 12 months, 12–24 months, or enrollment to 24 months) against the swine influenza viruses in a binary logistic regression model. Next, we examined risk factors for any increase in HI titer (using the participants’ greatest increase in titers, enrollment to 12 months, 12–24 months, or enrollment to 24 months) to the swine viruses through examining the entire spectrum of HI titer increase (e.g., no increase, 2-fold rise, 4-fold rise, 6-fold rise and 8-fold rise) through proportional odds modeling. We have found the proportional odds method to have greater power to detect important risk factor associations than more commonly used binary (yes or no) outcomes (*18*).

## Results

Among the 3,259 AHS persons contacted by telephone or mailing, 1,274 (39.1%) were considered eligible and were willing to participate. Among these, 803 (63.0%) attended enrollment sessions, granted informed consent, and were enrolled. After excluding 15 persons who self-reported accidental needle-stick with swine vaccine and another person with missing exposure information, 707 participants were classified as AHS swine-exposed and 80 as AHS nonswine-exposed. Enrollment data were compared with 79 nonswine-exposed University of Iowa controls (Table 1). More AHS swine-exposed participants were male than female and they also were older than those in the other 2 groups. The AHS nonswine-exposed participants were primarily women (96.3%); among these, 75.5% were spouses of AHS swine-exposed participants.

**Table 1:** Characteristics of study participants at enrollment*

Variables	AHS swine-exposed, no. (%), n = 707	AHS nonswine-exposed, no. (%), n = 80	University controls, no. (%), n = 79
Sex†			
Male	455 (64.4)	3 (3.8)	26 (32.9)
Female	252 (35.6)	77 (96.3)	53 (67.1)
Age group, y			
24–45	71 (10.0)	19 (23.8)	56 (70.9)
46–54	179 (25.3)	22 (27.5)	13 (16.5)
55–89	457 (64.6)	39 (48.8)	10 (12.7)
Mean age‡	56	51.1	35.3
Received influenza vaccine in the past 4 y			
Yes	392 (55.5)	43 (53.8)	44 (55.7)
No/unsure	315 (44.6)	37 (46.3)	35 (44.3)
Swine influenza vaccine in 1976*			
Yes	62 (8.8)	4 (5.0)	1 (1.3)
No	506 (71.6)	53 (66.3)	78 (98.7)
Unsure	132 (18.7)	22 (27.5)	0
Missing	7 (1.0)	1 (1.3)	0
Currently work with nursery or finishing swine			
Nursery swine	18 (2.6)	0	–
Finishing swine	126 (17.8)	0	–
Both	168 (23.8)	0	–
No	391 (55.3)	80 (100.0)	–
Missing	4 (0.6)	0	
Years worked in swine production			
Never	0	76 (95.0)	–
<1	1 (0.1)	0	–
1–4	10 (1.4)	0	–
5–10	38 (5.4)	0	–
>10	650 (91.9)	0	–
Missing	8 (1.1)	4 (5.0)	
On average, how often do you see or touch swine, other than the swine on the farm where you work?			
Never	270 (38.2)	49 (61.3)	–
Rarely	344 (48.7)	24 (30.0)	–
Monthly	27 (3.8)	0	–
Weekly	27 (3.8)	0	–
Every day	14 (2.0)	1 (1.3)	–
Missing	25 (3.5)	6 (7.5)	–
How long have you lived on this or other swine farm?			
Never	15 (2.1)	18 (22.5)	–
<1 y	1 (0.1)	1 (1.3)	–
1–4 ys	4 (0.6)	2 (2.5)	–
5–10 y	18 (2.6)	8 (10.0)	–
>10 y	636 (90.0)	42 (52.5)	–
Missing	33 (4.7)	9 (11.3)	–
Work in a slaughterhouse or meat processing plant			
Yes	4 (0.6)	2 (2.5)	–
No	674 (95.3)	75 (93.8)	–
Missing	29 (4.1)	3 (3.8)	–

*AHS, Agricultural Health Study; AHS swine-exposed, participants from the A HS who reported working in swine production; AHS nonswine-exposed, participants from the AHS who denied ever working in swine production (96.3% female and among these females 75.5% were spouses of the AHS swine–exposed); university controls, faculty, staff, and students from the University of Iowa who denied ever working in swine production. †Statistically significant considering a 95% confidence level by Fisher exact test for the 3 groups. ‡Statistically significant considering a 95% confidence level by analysis of variance test for the 3 groups.

During the 24 months of follow-up, 6 of the enrolled study participants died and 4 withdrew from the study. Among the remaining 788 volunteers, 709 (90%) participated in the 12-month follow-up encounters (632 AHS swine-exposed and 77 AHS nonswine-exposed). Serum samples were drawn from 658. Similarly, among the 788 AHS participants, 714 (91%) participated in the 24-month follow-up encounter (638 AHS swine-exposed, 75 AHS nonswine exposed). Serum samples were drawn from 654. Overall, 756 (96%) of 788 persons participated in at least 1 follow-up encounter, and 726 (92.1%) consented and provided at least 2 serum specimens.

### Self-Reported Exposures upon Enrollment

More than 50% of the participants reported receiving influenza vaccines during the 4 years before enrollment (Table 1). More than 90% of the AHS swine-exposed participants had worked with swine for >10 years, and 90.0% reported living on a swine farm for >10 years. Although AHS controls did not report direct swine exposure, 66.3% reported living on a swine farm, and 52.5% had done so for >10 years. Few participants had ever worked in the meat processing industry.

### Seroprevalence Findings upon Enrollment

The distribution of HI titers against swine influenza virus subtypes H1N1 and (H1N2) was different between groups. AHS swine-exposed participants had significantly higher titers against swine influenza subtypes H1N1 (geometric mean/percentage >40 = 9.7/12.4%, 6.5/5.0%, 5.1/0.0%) and H1N2 (geometric mean/percentage >40 = 12.9/20.2%, 7.5/6.3%, 5.6/1.3%), compared with AHS nonswine-exposed participants and university controls, respectively.

At enrollment, for both initial unconditional logistic regression (data not shown), and proportional odds modeling (Table 2), AHS swine-exposed and AHS nonswine-exposed participants had markedly higher distributions of antibody titers against both swine influenza viruses compared to university controls. For example, against swine influenza (H1N1), AHS swine-exposed persons had an adjusted odds ratio (OR) of 54.9. Interestingly, AHS nonswine-exposed persons also were at increased risk compared with university controls, with an adjusted OR of 28.2. Men had increased adjusted odds of elevated titers against both swine viruses compared with women. Receiving a flu shot in the past 4 years and having an antibody titer >40 against human influenza (H1N1) virus were important individual risk factors for elevated titers against swine influenza virus subtypes H1N1 and H1N2 viruses, respectively.

**Table 2:** Odds ratios for elevated hemagglutination inhibition assay antibodies (enrollment sera) against swine influenza virus using proportional odds modeling*

Variables	n	Swine (H1N1)			Swine (H1N2)	
		Unadjusted OR (95% CI)	Adjusted OR† (95% CI)		Unadjusted OR (95% CI)	Adjusted OR† (95% CI)
AHS swine-exposed	707	35.8 (8.7–146.8)	54.9 (13.0–232.6)		17.2 (7.9–37.7)	13.5 (6.1-29.7)
AHS nonswine-exposed	80	10.6 (2.4–47.5)	28.2 (6.1–130.1)		4.7 (1.9–11.4)	6.9 (2.8-17.2)
University controls	79	Ref	Ref		Ref	Ref
Age continuous	866	1.00 (0.99–1.01)	0.97 (0.96–0.98)		1.02 (1.01–1.03)	–
Sex						
Male	484	3.7 (2.8–4.9)	3.3 (2.4–4.5)		3.5 (2.7–4.5)	3.0 (2.3-4.0)
Female	382	Ref	Ref		Ref	Ref
Received flu shot in the past 4 y						
Yes	479	1.0 (0.8–1.3)	1.4 (1.1–1.9)		1.3 (1.0–1.7)	–
No/unsure	387	Ref	Ref		Ref	–
Human influenza (H1N1) (titer >40)						
Positive	347	1.1 (0.9–1.4)	–		1.6 (1.2–2.0)	1.8 (1.4-2.4)
Negative	519	Ref	–		Ref	Ref

*OR, odds ratio; CI, confidence interval; AHS, Agricultural Health Study; AHS swine-exposed, participants from the AHS who reported working in swine production; AHS nonswine-exposed, participants from the AHS who denied ever working in swine production, 94% were spouses of AHS swine-exposed; university controls, faculty, staff, and students from the University of Iowa who denied ever working in swine production. †Final multivariable models were designed that used a saturated model including all potential risk factors (see Methods) and manual backwards elimination.

### Self-Reported Exposures and Illness

Among the 726 study participants who provided serum samples in at least 1 follow-up encounter, 339 (46.7%) reported swine exposures during follow-up, 102 (14.0%) reporting never using gloves when working with animals, and 174 (24.0%) worked with >400 pigs on a farm during follow-up (Appendix Table). During the 24 months of follow-up, an influenza-like illness developed in 66 participants; they submitted 74 sets of self-collected nasal and gargle swab specimens. On average, specimens were collected within 2.8 days of symptom onset (range 0–7 days) and were received at the laboratory within 1.8 days of collection (range 1–5 days). Two of the study participants were culture positive for influenza B virus, and 22 were real-time RT-PCR and culture positive for influenza A virus. The hemagglutination genes of 21 of the 22 influenza A isolates were very similar to those from circulating human influenza (H3N2) viruses. However, complete genomic sequencing and phylogenetic analyses (data not shown) of 1 isolate (A/Iowa/CEID23/05) showed that this virus was a “triple reassortant” influenza (H1N1) virus (GenBank accession nos. DQ889682-DQ889689), with H1 HA, N1 NA, M, NP, and NS genes of classic swine influenza virus lineage, PB1 gene of human influenza virus lineage, and PA and PB2 genes of avian influenza virus lineage. Viruses of this genotype emerged among US swine in the late 1990s (*19*) following prior emergence of related human/swine/avian triple reassortant H3N2 and H1N2 subtypes among American pigs (*9*–*11*,*20*,*21*).

### Participant with Swine Influenza A Infection and Illness

The participant whose specimens yielded A/Iowa/CEID23/05 was a 50-year-old man who lived on a swine farm and was currently working with nursery and finishing swine. He self-reported having a sore throat, cough, runny/stuffed nose, and a measured oral temperature of 38.2°C at the time of culture. No headache, red/itchy eyes, body aches, chills, diarrhea, nausea/vomiting, or hoarseness were reported. He also reported exposure to sick swine (with symptoms of cough, runny nose, and/or poor food intake) during the 10 days before his illness. The isolation of A/Iowa/CEID23/2005, together with the prior recovery of genotypically related reassortant influenza (H1N1) and (H3N2) viruses from 2 people following apparent zoonotic transmission from pigs (*22*,*23*), indicates that viruses of human/swine/avian triple reassortant genotype can be human pathogens.

### Evidence for Influenza Infections during Follow-up

Like the enrollment serum samples, the 12-month and 24-month follow-up samples showed geometric mean titers that were elevated for the AHS swine-exposed compared with the AHS nonswine-exposed participants against swine influenza (H1N1) viruses (12 months 10.05, 7.18; 24 months 16.60, 8.71) and (H1N2) (12 months 11.64, 7.84; 24 months 10.14, 7.21). Although study participants’ sera were obtained at 12-month intervals and some infections were likely missed, we found considerable statistically significant evidence for recent influenza virus infection. Considering the 726 participants who donated serum at least twice and after examining each serum pair (enrollment to 12 months, 12 to 24 months, and enrollment to 24 months), 180 participants (25%) showed a >4-fold rise in antibodies against swine influenza (H1N1) virus, 37 (5%) against swine influenza (H1N2) virus, and 32 (4%) against human influenza (H1N1) virus at some time during the 24 months of follow-up (Table 3). There was more serologic activity against swine influenza (H1N1) during the 12- to 24-month follow-up period. However, among these same participants with rises in antibody titers, relatively few self-reported having influenza-like illness during the 24-month study period (Table 3).

**Table 3:** Serologic evidence for influenza infections during the 24 months of follow-up

Period	N	>4-fold increase							
		Swine influenza (H1N1)			Swine influenza (H1N2)			Human influenza (H1N1)	
		n	Reported ILI,* n (%)		n	Reported ILI,* n (%)		n	Reported ILI,* n (%)
Enrollment to 12-mo follow-up	658	26	3 (11.5)		17	7 (41.2)		10	1 (10)
12- to 24--mo follow-up	586	109	18 (16.5)		16	2 (12.5)		19	3 (15.8)
Enrollment to 24-mo follow-up	654	141	31 (22)		23	2 (8.7)		20	3 (15)
Any increase between pairs of serum samples†	726	180	38 (21.1)		37	9 (24.3)		32	4 (12.5)

*Percentage of the participants who demonstrated a ≥4-fold increase in titer who also self-reported an influenza-like illness (ILI) during follow–up. †From enrollment to 12 mo, 12 to 24 mo, or enrollment to 24 mo, among participants who permitted serum sample collections at least 2 times during the study.

After the paired serum samples were examined over time, AHS swine-exposed participants showed an increased risk for infection with swine influenza (H1N1) virus compared with AHS nonswine-exposed participants during the follow-up period (Appendix Table; OR 2.6, 95% confidence interval [CI] 1.3–5.4). However, identifying the specific exposure during follow-up that caused this increase in risk was elusive. We examined glove use, direct swine exposure during follow-up, the number of pigs exposed to during follow-up, and the type of direct swine exposure (nursery and finishing), as well as a history of influenza (human) vaccination and serologic changes in antibodies against human H1 influenza viruses. Although there were suggestions that these exposure variables were important, male sex was the strongest independent predictor of a 4-fold or any increase in titer over time. Similar analyses for increased titers against the swine influenza (H1N2) virus and stratifications of data by sex also failed to implicate a specific swine exposure as etiologic (data not shown).

## Discussion

Humans, pigs, and avian species are inextricably linked in influenza transmission. The 1918, 1957, and 1968 pandemic influenza viruses all had structural components from an avian influenza virus (*24*). During the 1918 pandemic, a concomitant epizootic of swine influenza spread across the US Midwest (*4*). Numerous anecdotal accounts described influenza-like illnesses developing in farmers and their families after contact with ill swine and of swine developing symptoms of swine influenza after contact with ill farmers (*3*). Since the 1918 pandemic, human influenza viruses have infected swine (*25*,*26*) and swine influenza viruses have occasionally caused recognized disease among humans (*27*). Swine influenza transmission is known to occur nonseasonally and sporadically in the US swine population. Approximately 25%–33% of 6- to 7-month-old finishing pigs and 45% of breeding pigs have antibodies to the classic swine influenza (H1N1) virus (*28*,*29*). Anticipating that the next pandemic influenza virus may be efficiently transmitted from swine to swine and between swine and humans, we examined risk factors for previous and incident swine influenza virus infections in humans as surrogates for pandemic virus risk among those occupationally exposed to swine.

Study results suggest that swine workers are at markedly increased risk for swine influenza virus infections. Swine workers (AHS swine-exposed) had >50 times the odds of elevated antibodies against the classic swine influenza (H1N1) virus and remarkably, the AHS nonswine-exposed (mostly spouses of swine-exposed participants) also were at increased risk, with >25 times the odds of influenza (H1N1) infection compared with truly nonexposed controls (university controls). These ratios suggest that the AHS nonswine-exposed participants acquired infection either through indirect exposure to swine (e.g., handling dirty laundry or exposure to other fomites), misclassification (did not report direct contact with swine but did occasionally enter a swine barn), or exposure to their spouses who were shedding swine influenza viruses. Although the latter explanation is likely a rare event, even spouses who reported never living on a swine farm had increased odds of elevated antibody titers (data not shown). These findings should be tempered with the acknowledgment that laboratory-based evidence for human-to-human transmission of swine influenza viruses is sparse in medical literature.

Consistent with our previous report (*7*), among the significant unadjusted risk factors, we found exposure to nursery pigs was associated with an increase in antibody titer over time to swine influenza (H1N1) virus (Appendix Table; OR 1.5, 95% CI 1.1–2.1), but being male was a stronger predictor. Among the participants who seroconverted to >1 of the swine viruses, <25% reported an influenza-like illness during the 2 years of follow-up, which suggested that most swine influenza virus infections are mild or subclinical. Among the 66 study participants with influenza-like illness who submitted 74 sets of gargle or nasal swab specimens through the US postal system, 22 cultures showed influenza A virus and 1 (4.5%) showed swine influenza virus.

This study has a number of limitations. Participation was voluntary, and participants might have been more likely to suffer zoonoses than their peers. Exposure data were collected through self-report, were unverified, and were subject to recall and other biases. University controls were younger than AHS participants and had substantially fewer years of life to come in contact with influenza viruses. Although age was selected in only 1 of the final multivariable models (Table 2), we checked for age difference confounding by forcing age in each of the other final multiviariate models, and the covariates presented in Tables 2 and 3 remained statistically significant (data not shown). As the study HI assays are strain dependent, a mismatch between circulating human or swine strains and those we used for the assays could have resulted in inaccurate estimates of risk.

Additionally, there was likely some confounding effect on antibodies against human influenza virus reacting in the HI assays against swine influenza virus. We attempted to control for potential cross-reactivity through statistical adjustments. However, these and the other demographic risk factor adjustments could have been inadequate to isolate swine exposure risk factors. Further, our detection of incident influenza virus infections was suboptimal. Paired sera were collected 12 months apart, which likely permitted some influenza virus infection to be missed. Also, because of the wide dispersal of study participants, we relied upon self-identification of influenza-like illness, self-collection of nasal and gargle specimens, and shipping of specimens by the US postal system, all likely reducing the probability of identifying influenza virus infections. Even so, we detected both serologic and culture evidence of incident swine influenza virus infections. This study is unique in that a large cohort of rural farmers, many with swine exposures, were prospectively followed for influenza-like illnesses. The aggregate study data clearly documents increased occupational risk of swine influenza virus infection for these workers and their nonswine-exposed spouses.

As our study data suggest, swine influenza virus infections in humans are often mild or subclinical; however, when detected they can be quite serious. Myers et al. recently reviewed the 50 cases in the medical literature and found the overall case-fatality rate to be 14% (*27*). Human clinical morbidity and mortality rates would likely be increased if a pandemic virus’s effect on rural communities were amplified by infection in swine herds. Thus, our data have important public health implications. With risk for infection so high and exposure so common, swine workers should be considered for special public health interventions (*1*). To our knowledge, there is no US national or state policy that offers swine workers priority access to annual influenza vaccines, pandemic vaccines, or influenza antivirals as part of influenza pandemic planning. These workers are also not considered a high priority for influenza surveillance efforts.

Protecting swine workers from influenza viruses will also benefit those with whom they have contact, namely family members, as well as the swine herds for which they care. Assuming an influenza virus may readily move among and between species, recent modeling studies have shown that such workers could accelerate an influenza epidemic among nonswine workers in their communities as much as 86% (*30*). Additionally, there is now extensive evidence for human influenza virus reassortment with swine and/or avian viruses in pigs (*9*–*11*,*19*–*21*,*25*,*26*). Encouraging swine workers to receive annual influenza vaccines will reduce their potential role in the genesis of novel influenza strains. Our study results corroborate the numerous arguments (*1*) that protecting swine workers from human and zoonotic influenza makes good public health sense.

## Supplementary Material

Appendix Table Risk factor analyses for an increase in antibody titer (at any point from enrollment to 24-month follow-up) against swine influenza virus among AHS participants*
