# Supplementary material for: Swine Workers and Swine Influenza Virus Infections
Source: Emerg Infect Dis. 2007 Dec;13(12):1871–8. doi: 10.3201/eid1312.061323 (PMC2876739; doi:10.3201/eid1312.061323)
Supplement: Appendix Table — Risk factor analyses for an increase in antibody titer (at any point from enrollment to 24-month follow-up) against swine influenza virus among AHS participants* [file 06-1323_appT-s1.pdf]

Appendix Table. Risk factor analyses for an increase in antibody titer (at any point from enrollment to 24–month follow-up) against swine influenza virus among AHS participants*

		Swine influenza (H1N1), 4-fold increase†			Swine influenza (H1N1), greatest increase‡					
Variables	Total sample	n (%)	Unadjusted OR (95% CI)	Adjusted OR (95% CI)	2-fold, n (%)	4-fold, n (%)	6-fold, n (%)	≥8-fold, n (%)	Unadjusted OR (95% CI)	Adjusted OR (95% CI)
Age (continuous)	726	180 (24.8)	1 (0.98–1.01)	–	226 (31.1)	128 (17.6)	37 (5.1)	15 (2.1)	1 (0.98–1.01)	–
Sex										
Male	419	129 (30.8)	2.2 (1.6–3.2)	2.2 (1.6–3.2)	144 (34.4)	96 (22.9)	23 (5.5)	10 (2.4)	2.3 (1.8–3.1)	2.3 (1.8–3.1)
Female	307	51 (16.6)	Ref	Ref	82 (26.7)	32 (10.4)	14 (4.6)	5 (1.6)	Ref	Ref
Exposure to swine during follow-up										
Swine exposure										
AHS swine-exposed at enrollment	650	171 (26.3)	2.6 (1.3–5.4)	–	204 (31.4)	123 (18.9)	33 (5.1)	15 (2.3)	2 (1.3–3.2)	–
AHS nonswine-exposed at enrollment	75	9 (12)	Ref	–	22 (29.3)	5 (6.7)	4 (5.3)	0 (0)	Ref	–
Exposure to swine during follow-up										
Exposed	339	92 (27.1)	1.3 (0.9–1.8)	–	113 (33.3)	69 (20.4)	15 (4.4)	8 (2.4)	1.3 (1–1.7)	–
Not exposed	387	88 (22.7)	Ref	–	113 (29.2)	59 (15.3)	22 (5.7)	7 (1.8)	Ref	–
Use of gloves when working with pigs										
Never wears gloves	102	25 (24.5)	1 (0.6–1.6)	–	23 (22.6)	19 (18.6)	4 (3.9)	2 (2)	0.7 (0.5–1.1)	–
Not exposed to pigs or exposed to pigs and wears gloves at least sometimes	624	155 (24.8)	Ref	–	203 (32.5)	109 (17.5)	33 (5.3)	13 (2.1)	Ref	–
No. of pigs on farm‡										
<400	463	120 (25.9)	2.2 (1.2–4.3)	–	149 (32.2)	85 (18.4)	25 (5.4)	10 (2.2)	1.9 (1.2–3)	–
≥400	174	48 (27.6)	2.4 (1.2–4.9)	–	51 (29.3)	36 (20.7)	8 (4.6)	4 (2.3)	1.9 (1.2–3.1)	–
Never directly exposed to pigs	89	12 (13.5)	Ref	–	26 (29.2)	7 (7.9)	4 (4.5)	1 (1.1)	Ref	–

Works with nursery pigs§										
Yes	559	131 (23.4)	1.3 (0.9–1.9)	–	164 (29.3)	93 (16.6)	29 (5.2)	9 (1.6)	1.5 (1.1–2.1)	–
No	723	178 (24.6)	Ref	–	225 (31.1)	126 (17.4)	37 (5.1)	15 (2.1)	Ref	–
Works with finishing pigs§										
Yes	262	73 (27.9)	1.3 (0.9–1.9)	–	91 (34.7)	54 (20.6)	12 (4.6)	7 (2.7)	1.4 (1.1–1.9)	–
No	461	105 (22.8)	Ref	–	134 (29.1)	72 (15.6)	25 (5.4)	8 (1.7)	Ref	–
Flu vaccination										
Vaccinated at least 1 of last 2 winters but otherwise not since 2001	168	38 (22.6)	0.8 (0.5–1.3)	–	58 (34.5)	22 (13.1)	14 (8.3)	2 (1.2)	1 (0.7–1.4)	–
Vaccinated at least 1 of last 2 winters and at least once other time since 2001	183	46 (25.1)	0.9 (0.6–1.5)	–	61 (33.3)	35 (19.1)	8 (4.4)	3 (1.6)	1 (0.7–1.4)	–
Vaccinated at least once since 2001 but not last winter	98	27 (27.6)	1.1 (0.6–1.8)	–	22 (22.5)	22 (22.5)	3 (3.1)	2 (2)	0.8 (0.5–1.3)	–
Vaccinated before 2001 but none since	50	9 (18)	0.6 (0.3–1.3)	–	16 (32)	6 (12)	2 (4)	1 (2)	0.7 (0.4–1.3)	–
No/unknown	227	60 (26.4)	Ref	–	69 (30.4)	43 (18.9)	10 (4.4)	7 (3.1)	Ref	–
Human antibody titer to (H1N1)										
≥4-fold increase	32	9 (28.1)	1.2 (0.5–2.6)	–	9 (28.1)	6 (18.8)	2 (6.3)	1 (3.1)	1.1 (0.6–2.1)	–
<4-fold increase	694	171 (24.6)	Ref	–	217 (31.3)	122 (17.6)	35 (5)	14 (2)	Ref	–

*AHS, Agricultural Health Study; OR, odds ratio; CI, confidence interval; Ref, Ref. Final multivariable models were designed using a saturated model with all the variables included in this table, and manual backwards elimination.

†Binary logistic regression modeling. a 4-fold increase at any time from enrollment to 24-month follow-up, among participants who permitted sera collections at least 2 times during the study.

‡Proportional odds modeling. The maximum increase in antibody titers from enrollment to 24-month follow–up, for each participant who permitted sera collections at least 2 times during the study. Grouping upper antibody titer levels when sparse, not to reject model assumption with the proportional odds score test. Greatest number of animals on farm during follow-up years.

§Based upon enrollment questionnaire. Summary counts that do not add up to 726 reflect missing data.
